# Noninvasive intracranial pressure assessment by optic nerve sheath diameter: Automated measurements as an alternative to clinician-performed measurements

**DOI:** 10.3389/fneur.2023.1064492

**Published:** 2023-02-01

**Authors:** Dag Ferner Netteland, Mads Aarhus, Erik Smistad, Else Charlotte Sandset, Llewellyn Padayachy, Eirik Helseth, Reidar Brekken

**Affiliations:** ^1^Department of Neurosurgery, Oslo University Hospital Ullevål, Oslo, Norway; ^2^Faculty of Medicine, University of Oslo, Oslo, Norway; ^3^Department of Health Research, Medical Technology, SINTEF, Trondheim, Norway; ^4^Department of Neurology, Oslo University Hospital Ullevål, Oslo, Norway; ^5^The Norwegian Air Ambulance Foundation, Oslo, Norway; ^6^Department of Neurosurgery, School of Medicine, Faculty of Health Sciences, University of Pretoria, Steve Biko Academic Hospital, Pretoria, South Africa

**Keywords:** optic nerve sheath diameter, ultrasound, intracranial pressure, traumatic brain injury, automated measurements, machine learning

## Abstract

**Introduction:**

Optic nerve sheath diameter (ONSD) has shown promise as a noninvasive parameter for estimating intracranial pressure (ICP). In this study, we evaluated a novel automated method of measuring the ONSD in transorbital ultrasound imaging.

**Methods:**

From adult traumatic brain injury (TBI) patients with invasive ICP monitoring, bedside manual ONSD measurements and ultrasound videos of the optic nerve sheath complex were simultaneously acquired. Automatic ONSD measurements were obtained by the processing of the ultrasound videos by a novel software based on a machine learning approach for segmentation of the optic nerve sheath. Agreement between manual and automated measurements, as well as their correlation to invasive ICP, was evaluated. Furthermore, the ability to distinguish dichotomized ICP for manual and automatic measurements of ONSD was compared, both for ICP dichotomized at ≥20 mmHg and at the 50th percentile (≥14 mmHg). Finally, we performed an exploratory subgroup analysis based on the software's judgment of optic nerve axis alignment to elucidate the reasons for variation in the agreement between automatic and manual measurements.

**Results:**

A total of 43 ultrasound examinations were performed on 25 adult patients with TBI, resulting in 86 image sequences covering the right and left eyes. The median pairwise difference between automatically and manually measured ONSD was 0.06 mm (IQR −0.44 to 0.38 mm; *p* = 0.80). The manually measured ONSD showed a positive correlation with ICP, while automatically measured ONSD showed a trend toward, but not a statistically significant correlation with ICP. When examining the ability to distinguish dichotomized ICP, manual and automatic measurements performed with similar accuracy both for an ICP cutoff at 20 mmHg (manual: AUC 0.74, 95% CI 0.58–0.88; automatic: AUC 0.83, 95% CI 0.66–0.93) and for an ICP cutoff at 14 mmHg (manual: AUC 0.70, 95% CI 0.52–0.85; automatic: AUC 0.68, 95% CI 0.48–0.83). In the exploratory subgroup analysis, we found that the agreement between measurements was higher in the subgroup where the automatic software evaluated the optic nerve axis alignment as good as compared to intermediate/poor.

**Conclusion:**

The novel automated method of measuring the ONSD on the ultrasound videos using segmentation of the optic nerve sheath showed a reasonable agreement with manual measurements and performed equally well in distinguishing high and low ICP.

## Introduction

In severe traumatic brain injury (TBI), assessment and control of intracranial pressure (ICP) are a pivotal part of standard patient management (1). Invasive measurement of ICP remains today's standard (2), despite certain limitations (3). From a global perspective, its demands on resources limit the availability for a proportion of patients with TBI worldwide. In addition, its invasive nature makes it unavailable in the prehospital setting or as an initial diagnostic triage tool. Based on this, a quest to develop a quick, reliable, and cost-effective noninvasive method for the assessment of ICP is warranted.

**Figure 1:**
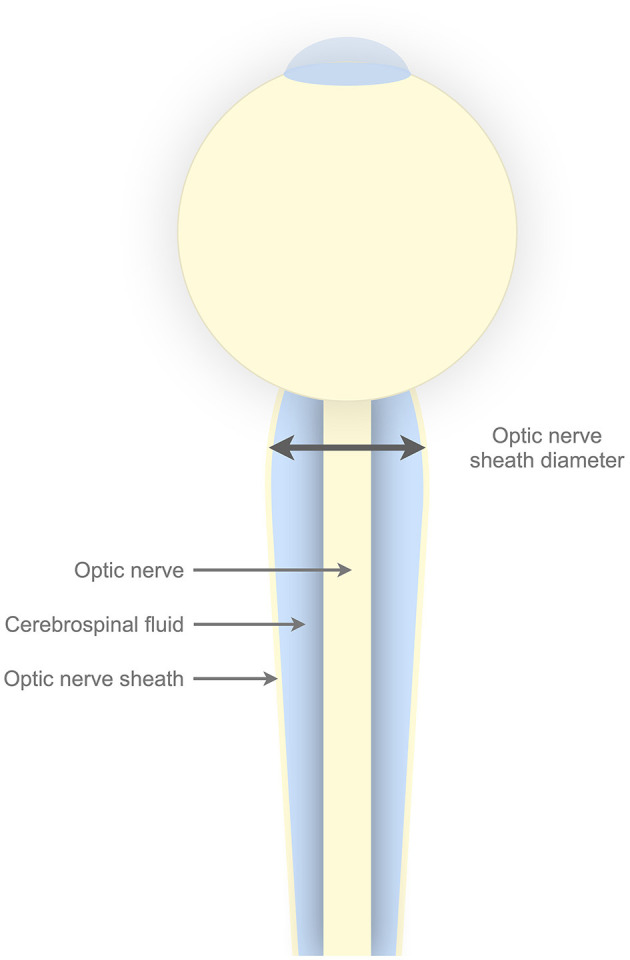
**Illustrative figure of the optic nerve sheath complex**. The optic nerve sheath envelopes the cerebrospinal fluid. The cerebrospinal fluid surrounding the optic nerve is in direct communication with intracranial cerebrospinal fluid. Horisontal line with arrows in both ends illustrates the optic nerve sheath diameter, which by convention is measured 3 mm posterior to the lamina cribrosa.

Optic nerve sheath diameter (ONSD) is a noninvasive parameter that has shown a promising association with ICP in previous studies (4–8). The parameter is made attractive by the fact that the cerebrospinal fluid (CSF) enveloped by the optic nerve sheath is in direct communication with the intracranial CSF (Figure 1). Therefore, the increases in ICP have the ability to distend the optic nerve sheath. The sheath can be visualized, and its diameter measured by ultrasound, making the method available bedside.

Bedside measurements of the ONSD do however remain operator-dependent, with inter- and intra-observer variability reported in the current literature (9–11). Automated measurements could potentially alleviate the need for operator skill and challenges with observer variability in determining the ONSD.

In this study, we evaluated a novel method of automated measurements of the ONSD and compared it against manual bedside measurements by an experienced ultrasound operator. Furthermore, we compared the correlation to invasively measured ICP and the ability to distinguish dichotomized ICP in automated and manual measurements.

## Methods

### Study design and data sources

In this study comparing manual and automated ONSD measurements, we obtained both ultrasound images and videos of the optic nerve sheath complex from adult patients with TBI admitted to a neuro-intensive care unit with invasive ICP monitoring. Manual bedside ONSD measurements were performed by an experienced ultrasound operator. At the time of manual measurements, videos of the optic nerve sheath were also obtained. Videos were processed remotely by a software for automated ONSD measurement. Invasively measured ICP was recorded at the time of image acquisition. Patients were examined by transorbital ultrasound and included in the study, as per the availability of the ultrasound operator.

Patients included in the study were treated according to our standard institutional TBI management protocol, concordant with brain trauma foundation guidelines (1), and inclusion in the study did not interfere with this management. In accordance with the Declaration of Helsinki (12), proxy consent was obtained for unconscious patients at the time of inclusion. If the patient regained the ability to give informed consent, this was obtained from the patient at a later stage. The study was approved by the Regional Committee for Medical and Health Research Ethics South East Norway (2018/136).

### Patient inclusion

Adult patients (≥18 years old) with head CT scan findings consistent with TBI who were admitted to the neuro-intensive care unit at Oslo University Hospital Ullevål with invasive ICP monitoring were eligible for inclusion. In patients with intraparenchymal ICP sensors, ultrasound datasets were acquired within 7 days of implantation to avoid significant drift from zero in the invasively measured ICP readings (13). When invasive measurement was performed via an external ventricular drain, no limitation to the in situ time was adhered to.

Patients with unilateral or bilateral injuries to the orbital region and pregnant patients were excluded from the study.

### Data acquisition and processing

Transorbital ultrasound examination was performed by an experienced operator using a commercially available ultrasound scanner (Philips Epiq 5G, Philips Healthcare, Andover, Massachusets, USA) with a linear array probe. Established safety margins of ophthalmic ultrasound imaging were adhered to, with a mechanical index <0.23. Serial measurements in same patient were obtained at opportunity.

Manual measurements were performed bedside on both eyes. The ONSD was measured 3 mm posterior to the lamina cribrosa of the sclera in one plane by the operator.

Automated measurements were based on the 10 second videos of the optic nerve sheath complex from both eyes. These videos were obtained directly after manual bedside diameter measurements were made for each eye. When recording the video, the probe was held still with the optic nerve sheath in focus. Deidentified ultrasound videos were exported at regular intervals for external processing by Nisonic AS (Trondheim, Norway), using a desktop version of the Nisonic P-100 software (version 2.0.0.13). The software is developed to perform real-time automated analysis of ONSD from ultrasound video sequences, based on a machine learning approach for segmentation of the nerve sheath. The determined value for each eye represents the 75th percentile of the estimated diameters for the entire ultrasound image sequence (10 seconds).

Invasive ICP was measured using either a parenchymal microsensor (Codman microsensor, Johnson and Johnson, Raynham, Massachusetts or Raumedic Neurovent-P ICP sensor, Raumedic AG, Muchberg, Germany) or a ventricular catheter and was recorded bedside at the time of ultrasound examination.

### Data analysis

The endpoints of the study were (1) agreement between manual and automatic ONSD measurements, (2) correlation between ONSD and ICP in manual vs. automatic diameter measurements, and (3) the ability of ONSD to distinguish dichotomized ICP in manual vs. automatic diameter measurements.

The pairwise difference between manual and automatic measurements of ONSD was evaluated using the Wilcoxon signed-rank test. Median pairwise difference is reported with interquartile range (IQR) as well as the root mean square error (RMSE).

Correlations between manual and automatic ONSD, manual ONSD and invasive ICP, and automatic ONSD and invasive ICP, were explored using the Pearson correlation coefficient with 95% confidence intervals (CIs).

To evaluate the ability of automatic and manual ONSD to distinguish between high and low ICP, cases were dichotomized by ICP ≥20 mmHg and ICP <20 mmHg. Median and IQR ONSD are reported for each group and compared using the Mann–Whitney *U* test, as well as receiver operating characteristic (ROC) curves with the associated area under curve (AUC) including 95% CIs. To compensate for a low number of cases with ICP ≥20 mmHg, we also performed the analyses with patients dichotomized by the 50th percentile of ICP.

Finally, to elucidate the reasons for variation in the agreement between automatically and manually measured ONSD in the material, we performed an exploratory subgroup analysis based on the automatic software's judgment of the optic nerve axis alignment in the image sequences. Optimal alignment is considered to be present when the optic nerve is centered in the image and the axis is perpendicular to the ultrasound transducer. The software indicates this evaluation by color codes, where green annotates good alignment, yellow indicates intermediate alignment and red indicates poor alignment of the optic nerve axis in the image sequence.

All statistical analyses were performed using MATLAB (R2022a, Mathworks Inc, MA, US), and a *p*-value of <0.05 was considered significant.

## Results

### Patient characteristics

A total of 25 patients with TBI were included in the study; 18 (72%) patients were men, and seven (28%) patients were women. The median age was 59 years (IQR 34.5–67.5 years). All patients sustained a blunt head injury, and the most common mechanism of injury was falls (40%) followed by motor vehicle accidents (16%) and sports and recreational injuries (16%). The median initial GCS score (14) was 6 (IQR 3–11.5). All patients were intubated and sedated at the time of examination.

### Ultrasound examinations of the optic nerve sheath

A total of 43 ultrasound examinations were performed on 25 patients, resulting in 86 image sequences covering the right and left eyes. Serial ultrasound examinations were performed in 12/24 (50%) of patients, and the median number of examinations in those with serial examinations was 2 (range 2–4).

The software failed to automatically segment and thereby give results for the ONSD in 3/86 (3%) of the image sequences.

In two examinations (same patient), numerical values for invasive ICP were missing. However, there were clear clinical (e.g., bilateral dilated pupils) and radiological findings consistent with high ICP. These examinations were included in the analysis of agreement between manual and automatic measurements and in the analysis of the ability of optic nerve sheath measurements to distinguish low and high ICP groups, where these examinations were annotated “high ICP.” They were however not included in the analyses of the correlation of ONSD to invasively measured ICP.

### Agreement between manual and automatic optic nerve sheath diameter measurements

**Figure 2:**
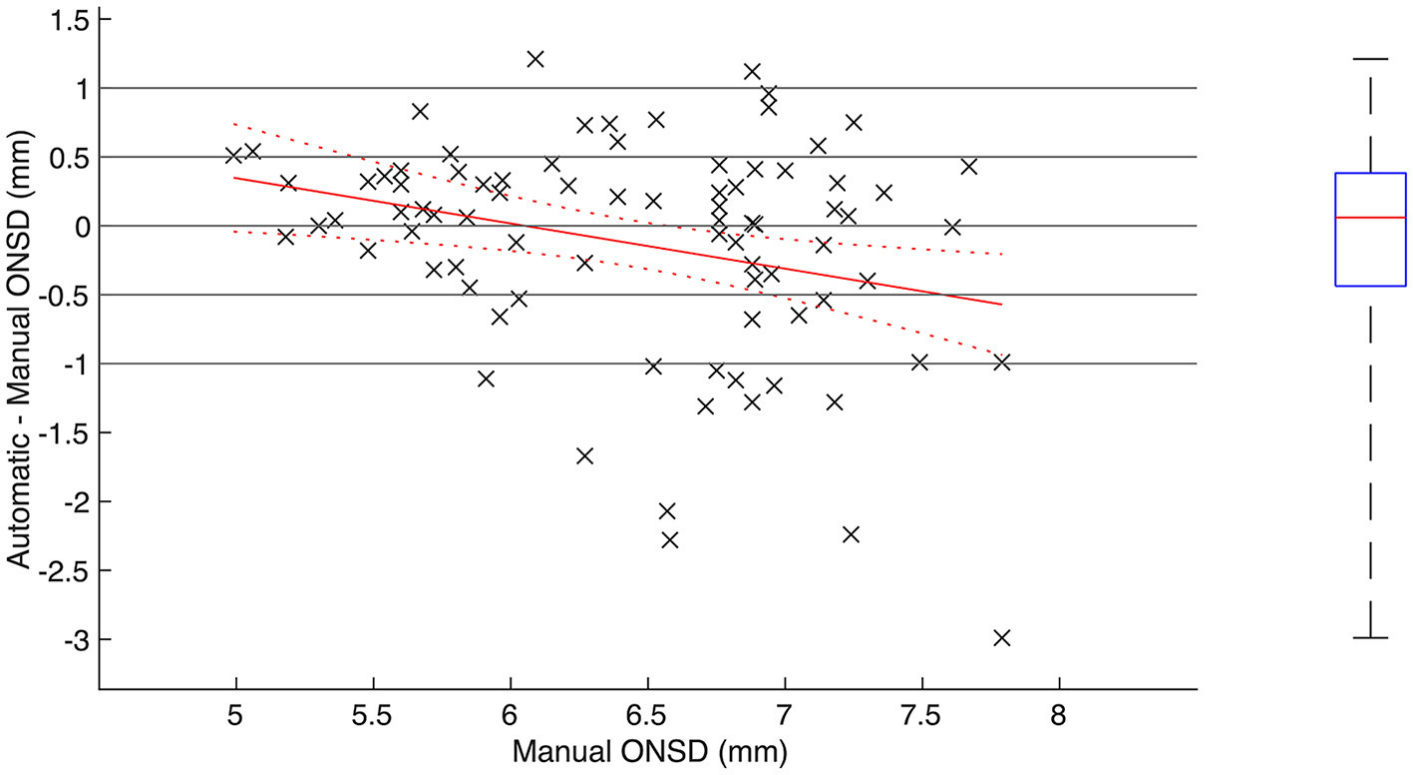
**Agreement between automatically and manually measured optic nerve sheath diameter. (Left)** Scatterplot including regression line and associated confidence bounds showing pairwise difference between automatically and manually measured ONSD plotted against manually measured ONSD. **(Right)** Boxplot showing median, IQR and range of pairwise difference.

In the 83 image sequences where the automatic segmentation software provided measurements for the ONSD, the median pairwise difference between automatically and manually measured ONSD was 0.06 mm (IQR −0.44 to 0.38; *p* = 0.80), and the RMSE was 0.80 mm. In 59% (49/83) of the image sequences, the difference was within ±0.5 mm, and for 82% (68/83) of the image sequences, the difference was within ±1.0 mm. In 16% (13/83), the automated method underestimated the ONSD by more than 1 mm, and in 2% (2/83), the ONSD was overestimated by more than 1 mm (Figure 2). There was a positive correlation between automatically and manually measured ONSD (*R* = 0.52, 95% CI 0.35–0.66; *p* < 0.001).

As illustrated in Figure 2, there was a conditional bias in the automatic measurements toward overestimating low ONSD and underestimating high ONSD (*R* = −0.29, 95% CI −0.47 to −0.08; *p* < 0.01). For manually measured ONSD below 6.4 mm (*N* = 38), there was a positive correlation between automatically and manually measured ONSD (*R* = 0.52, 95% CI 0.24–0.72; *p* < 0.001), and the median pairwise difference was 0.23 mm (IQR −0.12 to 0.40; *p* = 0.05). For manually measured ONSD greater or equal to 6.4 mm (*N* = 45), no statistically significant correlation could be proven (*R* = 0.24, 95% CI −0.06 to 0.50; *p* = 0.11), and the median pairwise difference was −0.06 mm (IQR −1.00 to 0.29; *p* = 0.08). The RMSE for ONSD <6.4 and ≥ 6.4 mm was 0.54 and 0.97 mm, respectively.

### Correlation between manual and automatic optic nerve sheath diameters and ICP

**Figure 3:**
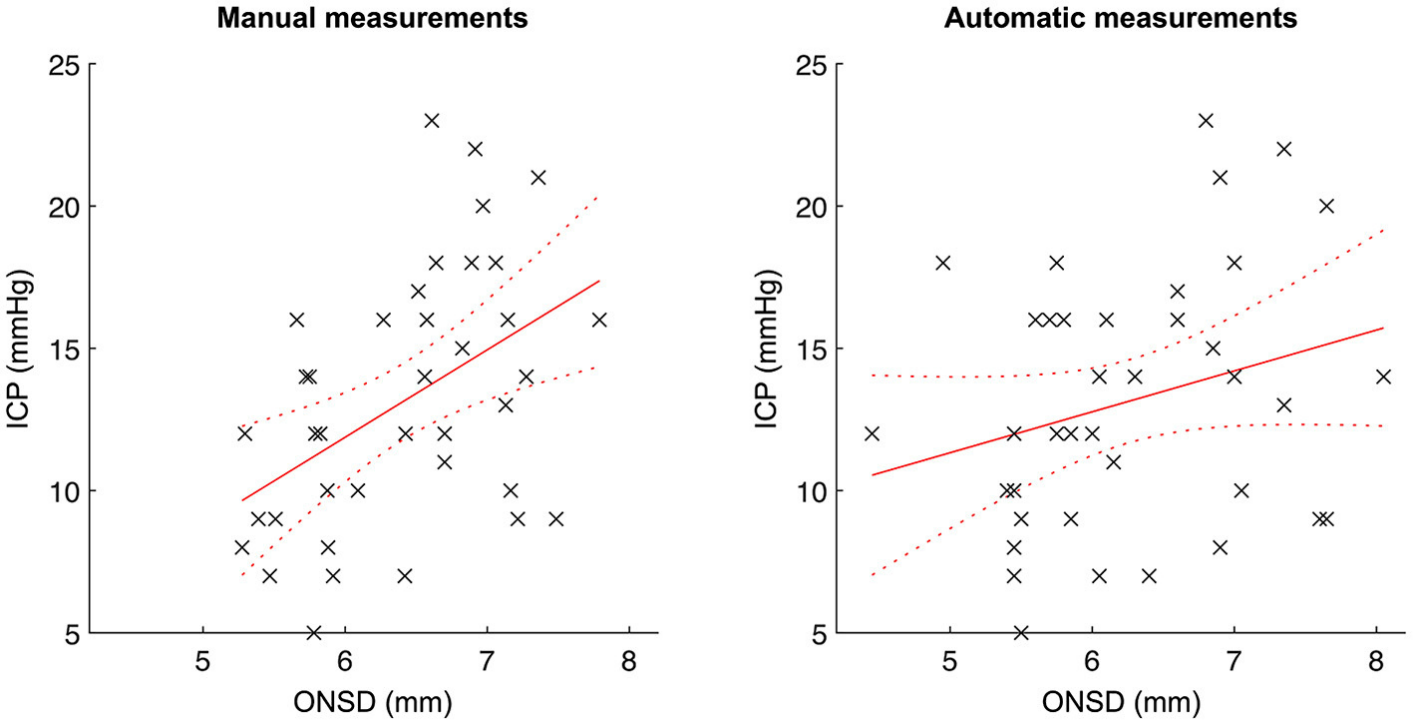
**Correlation between automatically/manually measured optic nerve sheath diameters and ICP**. Scatterplots with linear regression lines and associated confidence bounds for manual ONSD measurements **(left)** and automatic ONSD measurements **(right)**.

The correlation between mean ONSD (average between the left and right eyes for each examination) and ICP was calculated for all examinations where ONSD was automatically measured for both eyes, except for the two examinations where the numerical ICP values were lacking (*n* = 38; Figure 3). The manually measured ONSD showed a positive correlation with ICP (*R* = 0.47, 95% CI 0.17–0.68; *p* = 0.003). For automatically measured ONSD, we observed a trend toward, but not a statistically significant correlation with ICP (*R* = 0.27, 95% CI −0.06–0.54; *p* = 0.11).

### Ability of manual and automatic diameter to distinguish between dichotomized ICP

**Figure 4:**
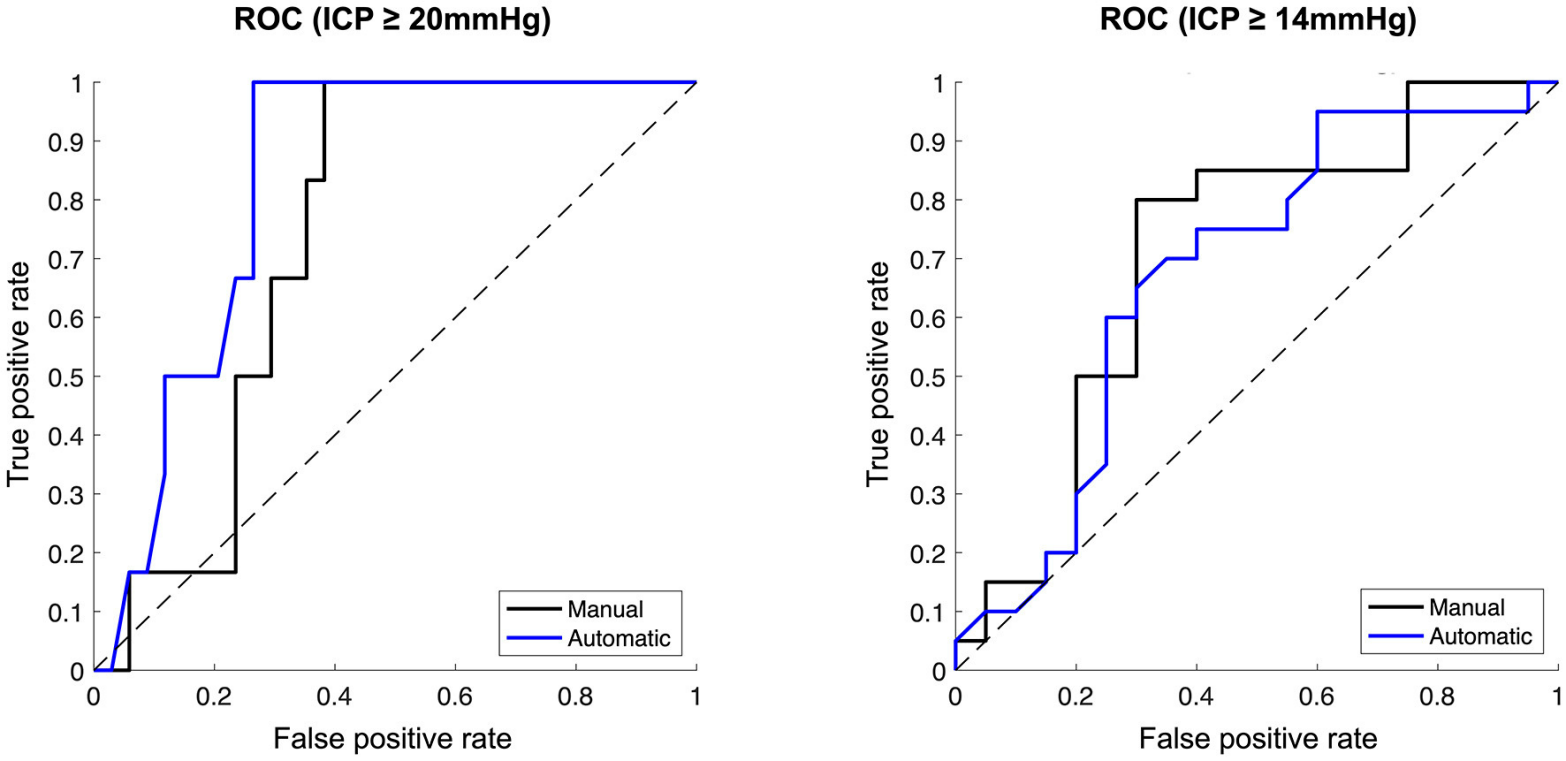
**Ability of manually vs. automatically measured optic nerve sheath diameter to distinguish dichotomised ICP**. Receiver operator characteristic curves. **(Left)** ICP dichotomised at ≥20 mmHg. **(Right)** ICP dichotomised at ≥14 mmHg. Manual ONSD measurements represented by black lines, automatic ONSD measurements represented by blue lines.

For an ICP threshold of 20 mmHg (low ICP<20 mmHg, *N* = 34; high ICP≥20 mmHg, *N* = 6), the median ONSD was significantly higher both for the manual (6.42 vs. 6.84 mm; *p* = 0.03) and for the automatic measurements (6.03 vs. 7.03 mm; *p* = 0.01) in the high ICP group. The AUC was 0.74 (95% CI 0.58–0.88) for the manually measured ONSD and 0.83 (95% CI 0.66–0.93) for the automatically measured ONSD (Figure 4).

Analysis by ICP threshold of 14 mmHg was subsequently performed to obtain balanced group sizes (low ICP < 14 mmHg, *N* = 20; high ICP ≥ 14 mmHg, *N* = 20). Here, the median ONSD was also significantly higher in the high ICP group both for the manual (6.70 vs. 5.90 mm; *p* = 0.01) and the automatic measurements (6.67 vs. 5.85 mm, *p* = 0.03). AUC was 0.70 (95% CI 0.52–0.85) for the manually measured ONSD and 0.68 (95% CI 0.48–0.83) for the automatically measured ONSD (Figure 4).

### Agreement between automatically and manually measured optic nerve sheath diameter by subgroups of the automatic software's judgment of optic nerve axis quality in the image sequences

**Figure 5:**
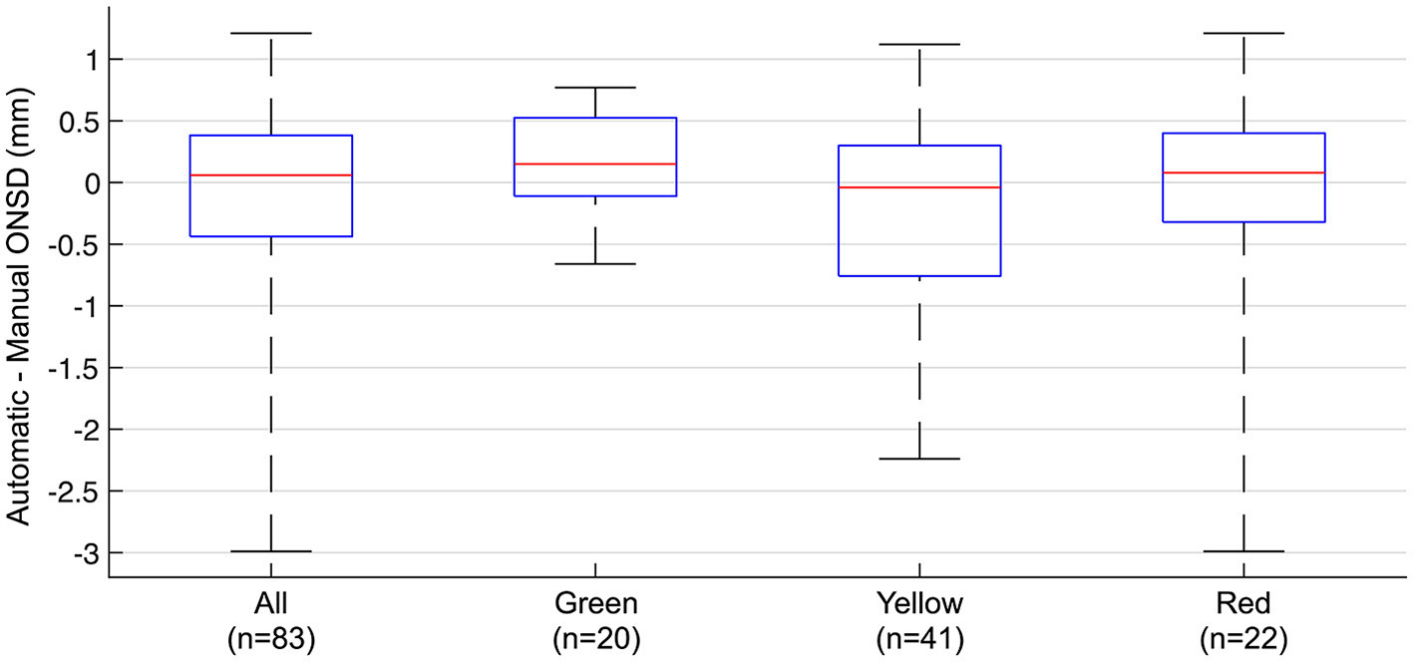
**Agreement between automatically and manually measured optic nerve sheath diameter by subgroups of the automatic software's judgement of optic nerve axis alignment in the image sequences**. Pairwise difference between automatically and manually measured ONSD in all measurements and subgroups. Box plots showing median, IQR, and range. Green, yellow, and red represents the automatic software's judgement of the optic nerve axis in the image sequences.

In the subgroup analysis by the automatic software's judgment of optic nerve axis alignment, the median pairwise difference between automatically and manually measured ONSD was 0.15 (IQR −0.11 to 0.53 mm; *p* = 0.12), −0.04 (IQR −0.76 to 0.30 mm; *p* = 0.20), and 0.08 mm (IQR −0.32 to 0.40; *p* = 0.85) in the green (*n* = 20), yellow (*n* = 41), and red (*n* = 22) groups, respectively (Figure 5). The RMSE was 0.47 mm in the green group, 0.81 mm in the yellow group, and 1.0 mm in the red group. The percentage of measurements where the difference was within ±0.5 mm was similar between all the groups (60% in green and 59% in yellow and red). However, in the green subgroup, the difference never exceeded ±1.0 mm, while in the yellow and red groups, the difference exceeded ±1.0 mm in 24 and 23% of image sequences.

## Discussion

In this study, we present the first results of a novel software for automatic measurement of the optic nerve sheath diameter on ultrasound videos using segmentation of the optic nerve sheath based on machine learning.

Overall, our results show a reasonable agreement between manual and automatic ONSD with a median pairwise difference of 0.06 mm. We found a statistically significant correlation between manual ONSD and ICP. For automatic measurements, we found a positive trend toward, but not a statistically significant correlation with ICP, which is likely related to an observed tendency of the automatic measurements to underestimate high diameters. When examining the ability to distinguish dichotomized ICP, manual and automatic measurements were performed with similar accuracy both for an ICP cutoff at 20 mmHg and for an ICP cutoff at 14 mmHg.

Both the novel software in its stage of development and the current study evaluating this software have some limitations. The study itself is ultimately limited by the number of ONSD measurements, especially the number of ONSD measurements in subjects with increased ICP. Regarding the novel software, we identified two potential sources of incorrect anatomical segmentation by visually reviewing the automatically segmented ultrasound images.

**Figure 6:**
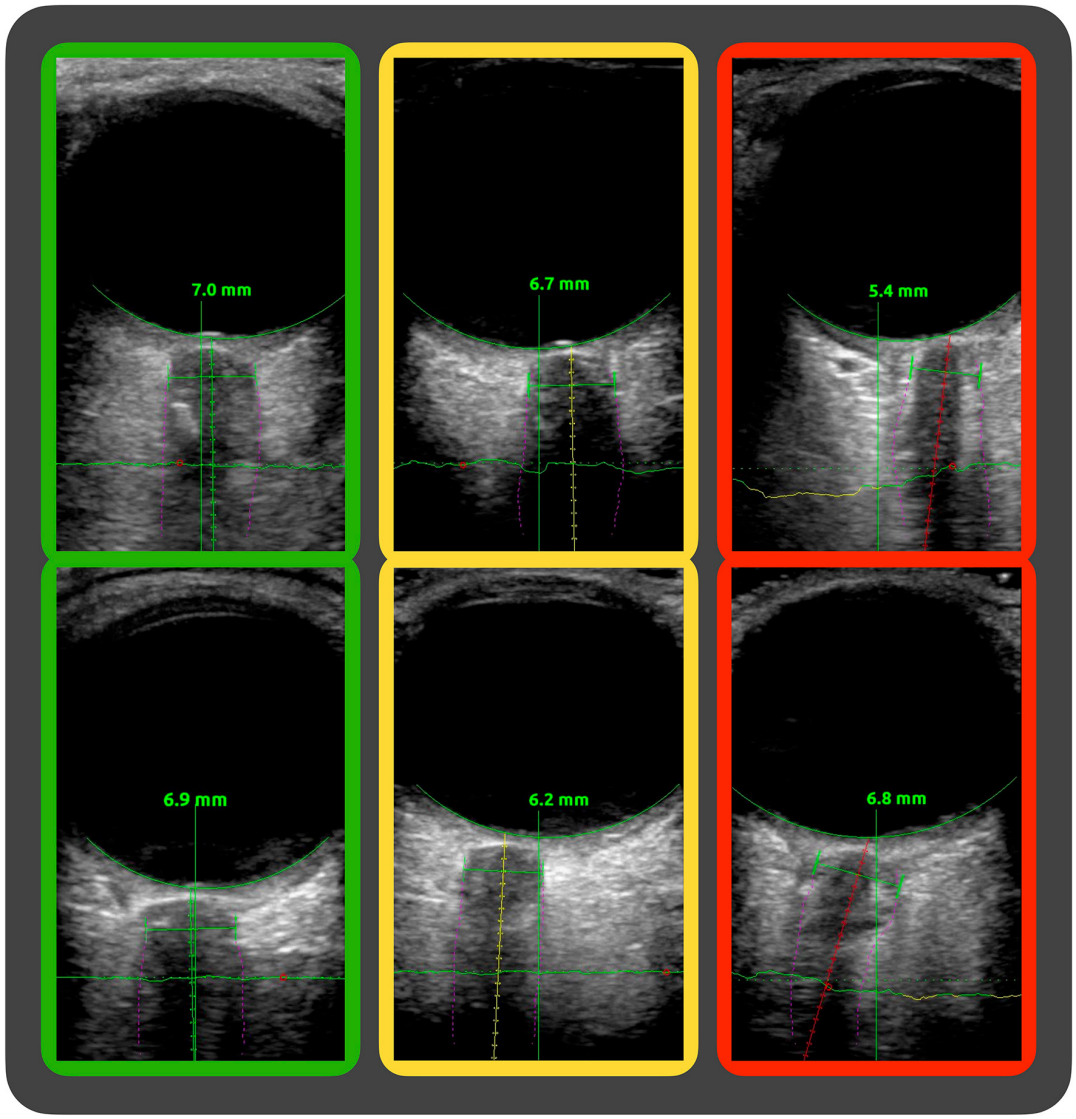
**Image examples of the software's evaluation of axis alignment**. Ultrasound images of the optic nerve sheath complex showing the software's segmentation (purple fine lines) of the nerve sheath and guidance system with differing evaluations of the axis alignment along the optic nerve. In the green, yellow, and red framed image, the software evaluation of axis alignment is good, intermediate, and poor, respectively.

First, we observed suboptimal anatomical axis alignment of the optic nerve in a subset of images, leading to skewed automatic measurements of the ONSD. The software evaluates the axis alignment and indicates this by green, yellow, and red color codes corresponding to good, intermediate, and poor axis alignments, respectively. This is part of the software's guidance feature meant to give the clinician aid in probe placement when used in its intended real-time bedside setting. In our exploratory analysis, we subgrouped the image sequences by the software's color codes of axis alignment and found that the agreement between manual and automatic measurements was highest in the green subgroup and decreased in the yellow and red subgroups. Image examples of the axis alignment with the software's color coding are given in Figure 6.

Second, we observed a small subset of images where the segmentation misinterpreted the optic nerve border as the optic nerve sheath.

This is a novel method based on machine learning developed for real-time segmentation of the optic nerve sheath complex point-of-care with software features to guide the clinician to optimal ultrasound probe alignment with the nerve sheath complex. In the present study, the software is evaluated by applying it retrospectively to ultrasound videos acquired by a commercially available ultrasound machine. This precludes the use of the guidance features while obtaining the ultrasound videos, and may, at least partially, account for the misalignment issues seen in some of the suboptimal anatomical delineations of the optic nerve sheaths by the software.

Furthermore, the automatic segmentation of the optic nerve sheath complex is based on machine learning, and the accuracy of the segmentation is expected to be dependent on the images on which the machine learning was based. This may offer an explanation as to why performance was poorer on high diameters, as machine learning to a large extent was based on images from the healthy volunteers with normal range diameters.

With the reduced performance seen with higher diameters, the method needs further development before being ready for clinical use, as accurate detection of high diameters is of crucial clinical importance. Nonetheless, the finding of a reasonable overall agreement between manual and automatic ONSDs is in our opinion an encouraging platform for future enhancements of the method. Feeding the algorithm with more pathological ICP values could be expected to improve performance also in the higher diameter range. Furthermore, implementing the software coupled to an ultrasound scanner to enable the measurements in its intended real-time, point-of-care environment could be expected to improve issues with misalignment with the optic nerve sheath complex.

Although still in need of increased accuracy, we believe that further development and research into the use of optic nerve sheath parameters may provide a reliable way of giving ICP estimates in the future. Its potential uses are multiple, and in a world where healthcare resources are finite, the development of a quick, easy, and cost-efficient way of estimating ICP may promise better care for patients with TBI and other neurological conditions globally.

## Conclusion

The novel automated method of measuring the optic nerve sheath diameter on ultrasound videos using segmentation of the optic nerve sheath showed a reasonable agreement with manual measurements and performed equally well in distinguishing high and low ICP. Further development of the method is warranted.

## Data availability statement

The raw data supporting the conclusions of this article will be made available by the authors, without undue reservation.

## Ethics statement

The studies involving human participants were reviewed and approved by the Regional Committee for Medical and Health Research Ethics South East Norway (2018/136). Written informed consent to participate in this study was provided by the patients/participants or patients/participants' legal guardian/next of kin.

## Author contributions

DFN: conceptualization, methodology, investigation, data curation, writing the original draft, writing, reviewing, editing, and funding acquisition. MA: conceptualization, writing, reviewing, editing, supervision, and funding acquisition. ES: methodology, software, data curation, writing, reviewing, and editing. ECS: conceptualization, methodology, writing, reviewing, editing, and supervision. LP: conceptualization, methodology, writing, reviewing, editing, supervision, and funding acquisition. EH: conceptualization, methodology, writing, reviewing, editing, supervision, project administration, and funding acquisition. RB: conceptualization, methodology, software, formal analysis, data curation, writing the original draft, reviewing, editing, supervision, and funding acquisition. All authors contributed to the article and approved the submitted version.
